# On the Power of Uncertainties in Microbial System Modeling: No Need To Hide Them Anymore

**DOI:** 10.1128/mSystems.00169-17

**Published:** 2017-12-05

**Authors:** Benoit Delahaye, Damien Eveillard, Nicholas Bouskill

**Affiliations:** a LS2N, UMR 6004, CNRS, Université de Nantes, ECN, IMTA, Nantes, France; b Lawrence Berkeley National Laboratory, Berkeley, California, USA; Mayo Clinic

**Keywords:** modeling, simulation, uncertainty

## Abstract

For decades, microbiologists have considered uncertainties as an undesired side effect of experimental protocols. As a consequence, standard microbial system modeling strives to hide uncertainties for the sake of deterministic understanding. However, recent studies have highlighted greater experimental variability than expected and emphasized uncertainties not as a weakness but as a necessary feature of complex microbial systems. We therefore advocate that biological uncertainties need to be considered foundational facets that must be incorporated in models. Not only will understanding these uncertainties improve our understanding and identification of microbial traits, it will also provide fundamental insights on microbial systems as a whole. Taking into account uncertainties within microbial models calls for new validation techniques. Formal verification already overcomes this shortcoming by proposing modeling frameworks and validation techniques dedicated to probabilistic models. However, further work remains to extract the full potential of such techniques in the context of microbial models. Herein, we demonstrate how statistical model checking can enhance the development of microbial models by building confidence in the estimation of critical parameters and through improved sensitivity analyses.

## Abstract (precis)

For decades, microbiologists have considered uncertainties as an undesired side effect of experimental protocols. As a consequence, standard microbial system modeling strives to hide uncertainties for the sake of deterministic understanding.

## INTRODUCTION

Since the work of Monod (1), simple biological modeling has been prominent in microbiology. Because of their experimental tractability and purported simplicity, microbial experimental systems have fostered the rise of several cross-scale modeling approaches from the gene to the population level, which have been extended to test ecoevolutionary hypotheses. These modeling approaches proposed and addressed foundational hypotheses that developed into new biological paradigms such as growth rate or identification of functional units. The first microbial models were driven by reductionist assumptions (e.g., intracellular quota combined with kinetics mimicking biochemistry rules) yet demonstrated remarkable predictive power for portraying the growth of microbes in simple systems such as chemostats (1). Similar quota assumptions were used to model phytoplankton physiology (2) and later for modeling simplified global ocean ecosystems (3).

Reductionist modeling approaches have generally been parameterized from data gleaned from laborious bench experiments. However, contemporary next-generation sequencing (NGS) approaches provide unprecedented characterization of the diversity of microbial communities, yet because feedbacks between biotic and abiotic systems are inherently nonlinear and complex, mathematical models of microbial guilds interacting with their environment are required. Current models have been developed along the lines of systems biology approaches (4, 5) or trait-based models (6). Furthermore, NGS provides a large amount of data that represent one experiment alongside its associated uncertainties. It is also worth noting that increasing the data set provides a concomitant increase in the number of uncertainties that must be considered.

Within microbial models, these uncertainties can be accounted for by machine learning techniques (see Libbrecht and Noble [7] for a review) that produce automatically predictive (deterministic) models from experimental measurements. Uncertainties are accounted for in the modeling process (e.g., via averaging) but are hidden in the model itself. Moreover, despite the great predictive power of such modelings, the resulting models are not necessarily biologically meaningful. In particular, once parameterized, a model could be overfit to a data set without reflecting emergent properties and precluding or reducing knowledge discovery. Considering that a single microbial system could produce several distinct data sets through several experimental approaches, several different models are built accordingly (8, 9). All corresponding models must then be investigated via automatic learning and verification techniques to take into account their common properties rather than considering each model in isolation (10). Conversely, other probabilistic modelings consider uncertainties but make the parameterization difficult or advocate the use of multiple models that are difficult to validate (11). Despite the aforementioned challenges, uncertainties must be accounted for and integrated into microbial modeling approaches. Such an issue remains a general problem across biology, and even in ecology despite a long tradition of dealing with quantitative uncertainties (12). Herein, as previously done in engineering (13), we advocate that the proper use of dedicated verification techniques could support efforts to capture the complexity of microbial systems within models by promoting a computational convergence on uncertainties rather than simple simulations. Below we present a short overview of current modeling approaches for studying microbial systems, after which we discuss the computational challenges that must be overcome to better take into account uncertainties and verify the resulting models.

## MICROBIAL STATE-OF-THE ART MODELINGS AND UNCERTAINTIES

Initial biological modeling efforts were inspired by models of physical systems and formalized using nonlinear ordinary differential equations representing dynamic behaviors of gene activity or molecular concentrations. Interactions within these models are driven by reaction rates associated with particular mechanistic behaviors such as Michaelis-Menten or Hill functions. The need to use formal methods to analyze such models has been discussed extensively in the last 15 years (see De Jong [14] for a state-of-the-art review or Fisher and Henzinger [15]). However, their application mainly results in a discretization of all interactions, such that the model becomes qualitative. An advantage in this context is that the resulting qualitative models are computationally scalable and do not need to incorporate an extensive number of parameters that are mostly out of experimental reach, including parameters that show clear sensitivity to experimental conditions (16). Nonetheless, even if such models are sufficient to represent microbial gene regulatory networks, they are generally not sufficient for modeling quantitative microbial behaviors as needed in the context of simulation of microbial populations and communities, and subsequently, biogeochemical processes.

In order to represent complex quantitative microbial community responses to environmental constraints, several studies postulate that the development of genome-scale metabolic models that consider the whole set of metabolic reactions within a microbial strain are necessary (17). In this context, the metabolic network consists of stoichiometric coefficients and mass balance constraints. Herein, the rates are reduced to fluxes that can be estimated via flux balance analysis (FBA) (18), which negates the need for specific kinetic parameterization yet provides quantitative predictions. FBA models are based on constraints, and solving the metabolic network analysis requires the consideration of boundary conditions for all metabolic fluxes that take place within the cell (see Bordbar et al. [19] for a review). In particular, recently, Basler et al. (20) have shown that those boundaries that reflect uncertainties are necessary to better understand intracellular metabolic flux distribution.

In order to simulate microbial communities, one can consider them at equilibrium by performing extensions of FBA (21, 22) or focus on dynamic quantitative behaviors by representing kinetics of several microbial populations via traits. These trait-based models use nonlinear differential equations and simplified complex dynamic behaviors with a single parameter. However, because of the inherent complexity of the population (23), estimating such a parameter is a difficult task without considering uncertainties and intensive statistical analyses. Despite several attempts to identify those traits from metagenomic experiments (24), estimating trait parameter values and automatically building predictive models from ecosystem experiments remain key challenges for the field.

## NEED FOR DEDICATED MODEL-CHECKING TECHNIQUES

**FIG 1:**
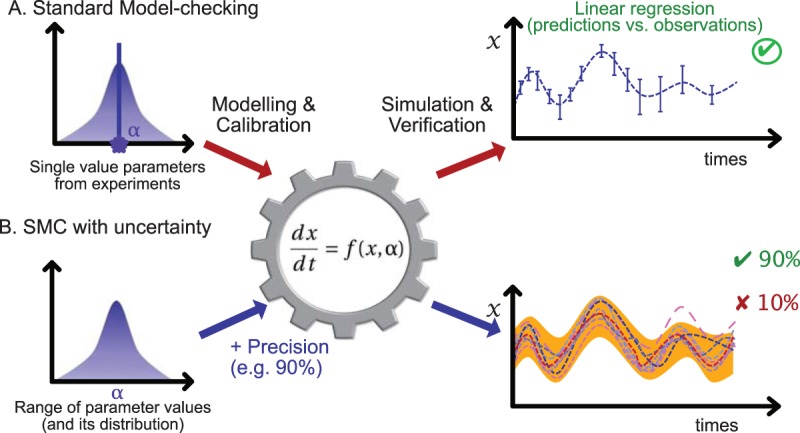
Illustration of model checking without and with uncertainties. Following a range of experiments, standard data analysis highlights the distribution of values for a given parameter α. (A) Along a standard model-checking protocol, one assumes a single value for α, usually the mean. Such a value is then used for model calibration, which allows a simulation. Simulation results are then compared with observations for the sake of model verification (e.g., usually via linear regression between prediction and observations). (B) An example of a model-checking protocol that considers uncertainties *per se*. Instead of considering a single parameter value, one considers a range of values and precision guarantees and performs a range of simulations accordingly (one per color). Altogether, this SMC approach validates the models while taking into account intrinsic uncertainties and guarantees the desired precision (90% here).

Following standard parameter estimation, whereby simulations satisfy the trends and trajectories of experimental data, models are usually tested through sensitivity analyses. Such analyses test the predictive accuracy of models across a wider range of parameter values. However, while such testing is commonly used in practice because of its computational scalability, sensitivity analysis does not provide a formal guarantee of the correctness of the model but rather a synthesis of extensive number of simulations. In several engineering fields, formal verification of models overcomes this shortcoming by performing model checking (25), which provides such guarantees. Unfortunately, because standard model checking was originally designed to study artificial systems, such as computer programs, it does not necessarily scale up to meet the demands of complexity of microbial systems, in particular when dealing with uncertainties. One must therefore combine model checking with sensitivity analyses, as fostered by recent statistical model-checking (SMC) methods (26–28). Like sensitivity analysis, SMC is based on simulations and executes the model several times to converge on a probability for a given property to be satisfied (see Fig. 1 for an illustration). In this case, the number of simulations that must be performed, and satisfied, is not arbitrarily fixed by the modeler but rather precomputed in order to ensure strong (formal) guarantees on the confidence and error levels of the analysis. Because SMC methods are based on simulations, they do not rely directly on the model structure (i.e., number of variables and constraints), only on the ability to run simulations, regardless of the formalization used (i.e., ordinary differential equations [ODEs], constraint-based, Boolean…). As a consequence, they are suitable for realistic modelings, ensuring strong formal guarantees (29) despite the complexity inherent to microbial systems.

## STATISTICAL MODEL CHECKING OF MICROBIAL MODELS

Because uncertainties are central to SMC methods, our hypothesis is that its use will be central for microbial model validation. SMC will indeed build formal confidence (trust) in the validation process while improving standard validation techniques. Standard validation is usually performed using a sufficiently large number of model simulations, but the precise number is left to the acumen of the modeler, which is not a satisfying guarantee with respect to the precision and correctness of the analyses. In contrast, the precision and correctness of SMC methods are formally certified by using statistical results to compute the required number of model simulations. Moreover, SMC methods are tailored for the analysis of models that incorporate uncertainties *per se* and therefore take into account parameter variations as standard characteristics of the models studied. Thus, two potential SMC applications in the context of microbial system validation could be emphasized.

### Model certification rather than sensitivity analysis.

Instead of fixing parameter values to their mean observed values and performing sensitivity analysis of one parameter at a time (Fig. 1A), we propose to embed the uncertainty of the parameter values into the models by assigning each parameter to a probability distribution based on its potential values informed by lab or field experiments (Fig. 1B). Consequently, a trait must then be considered a distribution on a range of values instead of a single value that represents multiple experiments. The distribution of values over a given range can thus be set as Gaussian, or as another distribution, in order to fit experimental uncertainties. In SMC, model simulations can be performed by picking parameter values within their attached distributions (i.e., by considering the variances of all parameters) and executing standard simulations. Following a simulation, deciding whether one prediction is valid can be done by computing the deviation of predicted values from existing observations. Since several simulations are performed (for several parameter values), SMC outputs a score representing the ratio of valid simulations. Thus, the SMC method performs a generalization of standard sensitivity analyses, not by analyzing the sensitivity of a single average simulation but by analyzing all feasible simulations and proposing general statistics of the whole; i.e., accurate statistical guarantees to perform predictive simulations while taking into account experimental uncertainties. By extension, considering uncertainties also allows us to certify all simulation behaviors (i.e., average of simulations) for the sake of model validation, rather than validating a single behavior (i.e., simulation of an average).

### Model parameter estimation with uncertainties.

**FIG 2:**
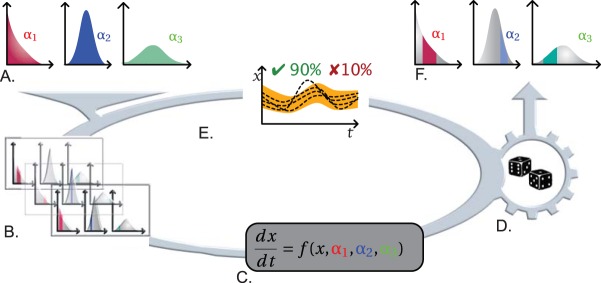
Statistical model checking (SMC) for model parameter estimation with uncertainties. (A and B) Considering the distribution of parameter values (A), SMC will perform a partition of the global range of parameter values (B). Notably, all parameters will be identified as a whole, instead of identifying each parameter independently from others as in standard parameterization techniques. (C and D) Probabilistic simulations are then performed for each of the “subranges” obtained. (E) Simulations of all models are then compared to experimental data for the sake of adequacy estimation. (F) Iterated several times, this protocol allows us to identify parameter subranges that, considered altogether, best fit the experimental data.

Model parameters are often difficult to measure because microbial communities encapsulate an array of trait data leading to wide ranges in parameter values. Moreover, identifying a distribution of parameter values, for instance, trait parameter values for models of microbe-mediated biogeochemical processes remains a difficult task. One could overcome this difficulty by considering a slight modification of the SMC paradigm to decipher the global set of parameters (with uncertainties) that best fit the experimental data (Fig. 2). While the global range of potential parameter values is often known, the aim here is to identify, within this range, the “ideal” parameter values (i.e., those that produce the highest rate of valid simulations). In this context, a partition of the global range of parameter values (alongside the variance) is produced (Fig. 2B), and simulations are performed for each of the obtained “subranges” (Fig. 2C). SMC can then provide a certified score for each of those subranges, which represent an evaluation of their adequacy with respect to experimental data (Fig. 2D and E). When performed on all subranges from the partition, this method allows us to identify the parameter subranges that best fit the experimental data.

## CONCLUSION

Uncertainties in parameter values are inherent to models built from experimental data. Instead of doing our best to rule out these uncertainties through deterministic modeling, we advocate that they should be incorporated into the models via the development of dedicated probabilistic modelings. Originally developed for software applications, statistical verification of such models will enhance the accuracy of model validation while also bringing formal evidence of the correctness of the approach. In addition, the future development of dedicated SMC methods will be the necessary steps to estimate parameter values with uncertainties, ensuring the satisfaction of desired properties, which represents the next methodological block for modeling quantitatively microbial systems from genes to ecosystems.
